# Morphine potentiates HIV infection and receptor expression in 3d brain organoids

**DOI:** 10.1007/s13365-026-01316-8

**Published:** 2026-05-18

**Authors:** Michael Swingler, Doga Tasdemir, Senem Cakir, Anna Bellizzi, Martina Donadoni, Ilker K. Sariyer

**Affiliations:** Department of Microbiology, Immunology, and Inflammation, Center for Neurovirology and Gene Editing, Temple University Lewis Katz School of Medicine, Philadelphia, PA USA

**Keywords:** Cerebral organoids, Opioids, HIV-1, Morphine, Virus, Infection

## Abstract

Opioid abuse is a major comorbidity of HIV, yet its direct effects on the brain remain unclear. Using iPSC-derived 3D human cerebral organoids (hCOs), we show that morphine directly upregulates HIV receptors CD4, CCR5, and CXCR4 in the absence of peripheral immune cells or a blood–brain barrier. This receptor induction drives a significant increase in HIV viral load within the CNS, revealing a brain-intrinsic mechanisms of opioid-mediated viral enhancement. These findings establish hCOs as a unique platform to investigate neuroHIV and provide critical insight into how opioids amplify CNS infection independently of peripheral factors.

## Introduction

Substance use, particularly opioid use, is a well-established comorbidity of human immunodeficiency virus (HIV) infection. Among people who inject drugs (PWID), 83% primarily use opioids (Degenhardt et al., 2023), and approximately 18% of PWID are estimated to be HIV-positive. Injection drug use accounts for 10% of new HIV infections globally and 25% of new infections outside sub-Saharan Africa (Degenhardt et al., 2017; People Who Inject Drugs (PWID), International Association of Providers of AIDS Care, n.d.).

Previous studies have shown that opioids such as morphine and fentanyl enhance HIV replication in macrophages and monocytes (Li et al. 2003; Murphy et al. 2019; Madhuravasal Krishnan et al. 2023) and upregulate HIV co-receptors CXCR4 and CCR5 in cell and rodent models (Miyagi et al. 2000; Guo et al. 2002; Steele et al. 2003; Gonek et al. 2018). However, these models lack the cellular complexity and three-dimensional structure of the human central nervous system (CNS).

Here, we employ human induced pluripotent stem cell (hiPSC)-derived cerebral organoids (hCOs) to investigate the effects of morphine on HIV replication and receptor expression in a complex 3D CNS-relevant model. Our hCOs contain neurons, oligodendrocytes, astrocytes, and microglia, and express the receptors necessary for HIV infection (Donadoni et al. 2024). These organoids support productive HIV infection, which can be suppressed by combination antiretroviral therapy (cART) (Donadoni et al. 2024). Using this system, we demonstrate that morphine directly induces expression of CD4, CCR5, and CXCR4 and significantly increases HIV viral load, even in the absence of peripheral immune cells or a blood–brain barrier. These findings reveal a CNS-specific mechanism by which opioids enhance HIV infection and highlight hCOs as a unique platform to study neuroHIV and opioid interactions in a human-relevant context.

## Results

### Morphine enhances HIV viral replication in 3D human cerebral organoids (hCOs)

**Fig. 1:**
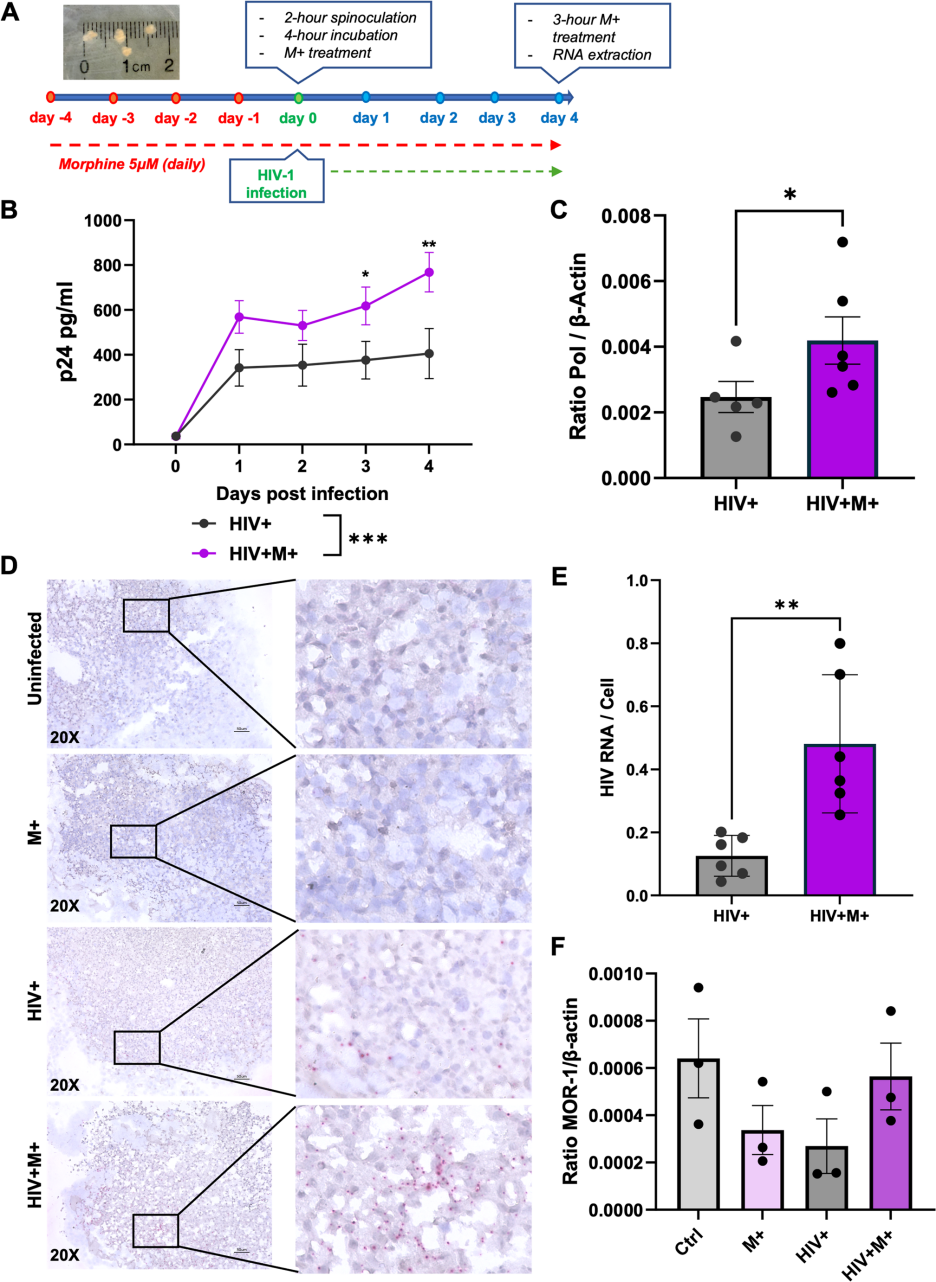
Morphine enhances HIV infection in human cerebral organoids (hCOs).**(A)** Experimental timeline and representative hCO image. hCOs were treated with 5 µM morphine for 4 days prior to HIV infection. Morphine treatment was resumed daily post-infection until collection. **(B)** p24 Gag ELISA (pg/mL) of supernatants from HIV + and HIV + M+ hCOs at days 0–4 post-infection. **(C)** RT-qPCR of HIV Pol RNA as a ratio to β-actin. **(D)** Representative 20X RNAscope images showing HIV RNA (red dots). **(E)** Quantification of HIV RNA per cell using ImageJ. **(F)** RT-qPCR of MOR-1 RNA as a ratio to β-actin. Statistical significance assessed by Student’s t-test and two-way ANOVA (*p* < 0.05)

hCOs were generated from hiPSCs using established protocols from our recent studies and others, resulting in an in vitro model of the human brain (Lancaster et al. 2013; Bellizzi et al. 2024; Donadoni et al. 2024; Birtele et al. 2025). These hCOs have previously been extensively characterized and shown to express neurons, astrocytes, oligodendrocytes, and microglia, and proved to be an effective model for HIV-1 infection (Donadoni et al. 2024). hCOs were treated with or without 5 µM morphine for 4 days prior to HIV infection. hCOs were then infected with HIV-1 as described in materials and methods, and morphine treatment was resumed that day if applicable. Infected and uninfected organoids were treated with or without morphine for additional 4 days, and 200 µL of media was saved daily for p24 ELISA. At day 0, low levels of p24 were present despite washing the hCOs of inoculum after infection. The p24 levels in HIV + and HIV + M+ hCOs increased everyday post infection as expected for the progressive viral replication. Interestingly, the p24 levels of HIV + M+ hCOs were significantly higher than those of HIV+ alone (*p* = 0.0005), most with the most significant differences at D3 (*p* = 0.0385) and D4 (*p* = 0.0034) post infection (Fig. 1B). To further characterize the effects of morphine on HIV infection, RNA samples from these hCOs were analyzed by RT-qPCR for HIV transcript Pol and β-actin. Consistent with the p24 findings, we see a significant increase in Pol expression in HIV + M+ hCOs compared to HIV+ alone (*p* = 0.0126) (Fig. 1C). No Pol was detected in uninfected samples (data not shown). Additionally, RNAscope staining for HIV was performed on uninfected or infected hCOs treated with and without morphine. Representative 20X images are shown in Fig. 1D. Intensity of viral RNA staining was quantified as HIV RNA dots/cell using ImageJ and data is shown as a bar graph (Fig. 1E). RNAscope again revealed a significantly greater staining intensity in HIV + M+ hCOs compared to HIV+ alone (*p* = 0.0093). This further confirms the increase of HIV viral load following morphine treatment in this system. Moreover, OPRM1 receptor expression was also assessed by qRT-PCR in the same samples. Interestingly, no significant differences in mu opioid receptor (MOR-1) expression were observed among the groups. (Fig. 1F).

## Morphine increases HIV receptor expression in hCOs

**Fig. 2:**
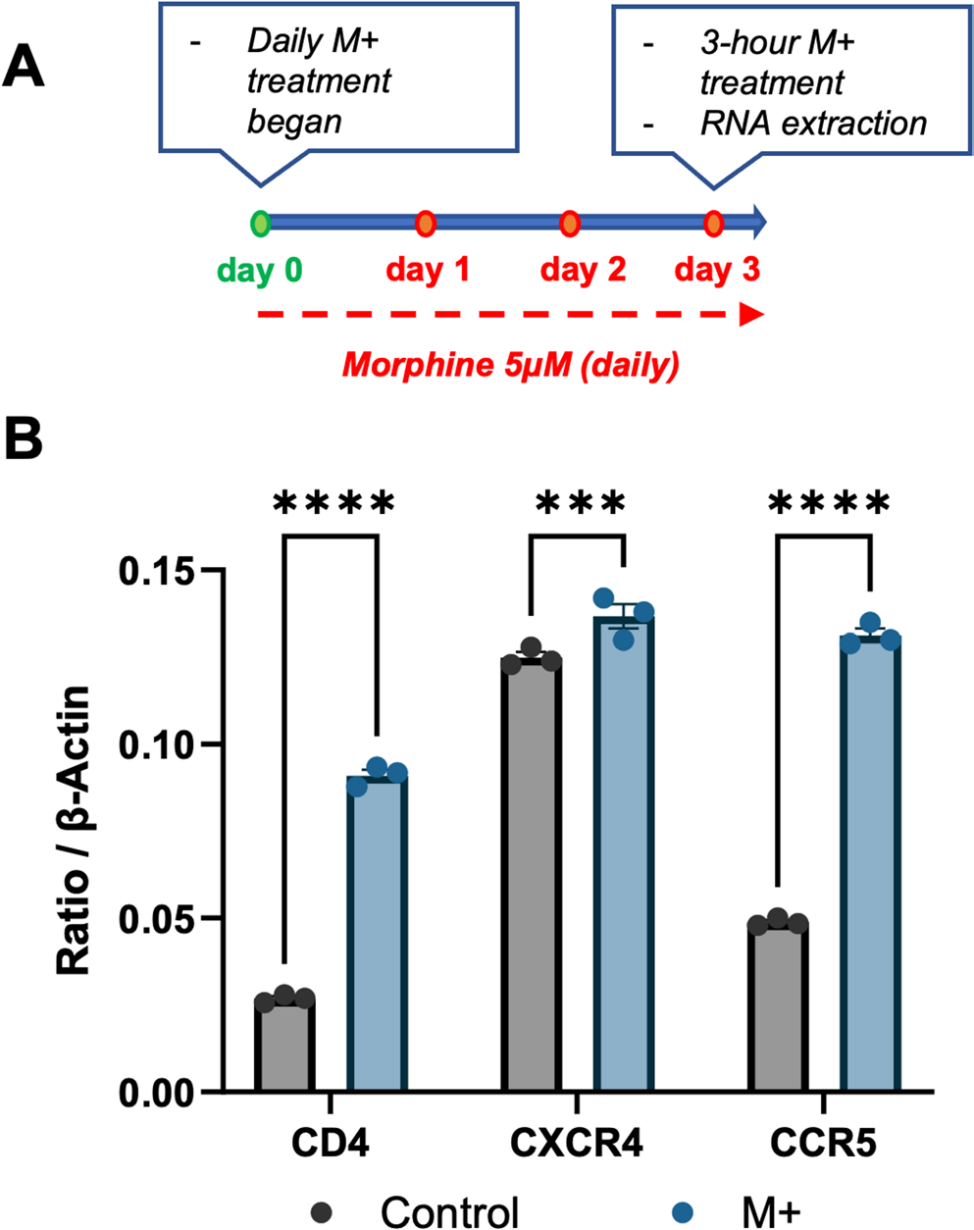
Morphine increases HIV receptor expression in hCOs.**(A)** Experimental timeline of morphine treatment. hCOs were exposed to 5 µM morphine for 3 days. **(B)** RT-qPCR analysis of HIV receptors CD4, CXCR4, and CCR5 graphed as ratio to β-actin. Statistical significance was determined by two-way ANOVA (*p* < 0.05)

After showing the effect of morphine on increasing HIV replication in hCOs, we sought to characterize its effect on HIV receptor and co-receptor expressions in this system prior to infection. hCOs were treated with 5 µM morphine daily for 3 days, after which RNA was extracted and RT-qPCR for HIV receptors CCR5, CXCR4, and CD4 were performed with β-actin as a reference gene (Fig. 2A). Morphine significantly increased CCR5 (*p* < 0.0001), and to a lesser extent, significantly increased CXCR4 (*p* = 0.0010) (Fig. 2B). Interestingly, morphine also resulted in a significant increase in CD4 expression (*p* < 0.0001). This data shows that morphine directly increases HIV receptor and co-receptor expressions in hCOs even in the absence of peripheral immune cells.

## Discussion

Studying the interaction between opioids and HIV infection in the human brain, and neuroHIV in general, has been notoriously difficult due to lack of adequate model systems. The rodent models available are not always translatable to humans due to genetic differences, and in vitro models have only explored 2D culture systems. Thus, hCOs prove to be a uniquely powerful tool for studying neuroHIV and other viral infections, as they express all neural cell types and form a complex structure that is representative of the human brain (Antonucci and Gehrke 2019; Swingler et al. 2023; Bellizzi et al. 2024; Donadoni et al. 2024). Morphine treatment of iPSC-derived hCOs significantly increased HIV viral load and replication, as demonstrated by p24 ELISA, RT-qPCR of HIV Pol, and RNAscope detection of viral RNA. These findings recapitulate prior observations in cell-based models while extending them to a multicellular neural system that better reflects the human brain microenvironment. Although HIV does not directly infect neurons, these cells are primary targets of opioids, raising important questions about indirect interactions and downstream effects. Our results highlight hCOs as a powerful platform to investigate opioid–neuroHIV interactions and their consequences.

While the hCOs in this study contain neurons, oligodendrocytes, astrocytes, and microglia, they still lack a blood brain barrier (BBB), peripheral immune components, and vasculature, all of which are crucial aspects of opioid use disorder and HIV (McCarthy et al. 2001; Watkins et al. 2007; Plein and Rittner 2018; Donadoni et al. 2024). Morphine and HIV promote infiltration of infected monocytes into the brain, contributing to increased viral burden. Both disrupt the BBB and facilitate entry of free virus and infected cells into the central nervous system. In our hCO model, which lacks a BBB and peripheral immune components, morphine still enhances HIV infection. This finding suggests that morphine may directly increase viral replication in the brain, independent of peripheral mechanisms.

hCOs were pretreated with morphine for four days prior to HIV infection to model opioid use preceding infection, as occurs in PWID. In parallel, hCOs were exposed to morphine for three days to assess HIV receptors expression. Morphine treatment significantly increased expression of CD4, CXCR4, and CCR5 (Fig. 2). CCR5 is the primary neurotropic HIV co-receptor, whereas CXCR4 is more commonly associated with T cells (Vallat et al. 1998). Despite this, baseline CXCR4 expression was higher than CCR5 in hCOs, consistent with our previous findings (Donadoni et al. 2024). Morphine-induced upregulation of the co-receptors CCR5 and CXCR4 has been previously characterized in macrophage cell types. Here, we recapitulate this effect in a model that includes diverse central nervous system cell populations, extending these findings to a more physiologically relevant context. (Guo et al. 2002; Li et al. 2003; Murphy et al. 2019; Madhuravasal Krishnan et al. 2023). We also observed a significant increase in CD4 expression, the primary receptor required for HIV entry. These findings suggest that opioid exposure may increase susceptibility to HIV infection by upregulating viral receptor expression prior to infection, while also directly enhancing viral burden in the brain.

Altogether, our data indicates that morphine directly amplifies HIV viral load by upregulating viral receptor expression in a complex 3D in vitro model of the human brain. This model provides a platform for future studies aimed at elucidating the mechanisms underlying opioid-mediated enhancement of neuroHIV in a human-relevant system.

## Materials and methods

### Cell culture and hCO generation

Human induced pluripotent stem cell (hiPSC) derived human cerebral organoids (hCOs) were generated using STEMdiff™ Cerebral Organoid Kit (STEMCELL TECHNOLOGIES, 08570), following manufacturer’s instructions with few modifications as we previously described (Bellizzi et al. 2024; Donadoni et al. 2024). hiPSCs were seeded in 96-Well Ultra-Low Attachment Round-Bottom plates (Millipore Sigma, CLS7007) in embryoid body (EB) seeding media at a density of 12,000 cells/well and cultured for 6 days. Following 6 days in EB seeding media, each EB was transferred into a single well of 24-Well Ultra-Low Attachment plates (Millipore Sigma, CLS3473) containing induction Medium. On day 8, each EB was embedded in 15 µL of Matrigel^®^ (CORNING, 354277) on organoid embedding sheets (STEMCELL TECHNOLOGIES, 08579). After Matrigel was allowed to polymerize, embedded EBs were transferred to 6-Well Ultra-Low Attachment plates (STEMCELL TECHNOLOGIES, 38071) containing expansion media. On day 12, EBs were switched to maturation media and placed on an orbital shaker and incubated at 37˚C with 5% CO_2_. A half-media change was performed every 3 to 4 days during and after maturation with identical conditions. hCOs were considered mature 50 days after initial seeding.

### Morphine treatment

Morphine (Sigma-Aldrich, M8777-50MG) was reconstituted in molecular grade H_2_O at a concentration of 10mM and frozen in single use aliquots at −80˚C. Morphine was diluted to working concentration and added to culture system to reach the desired final concentration of 5 µM. hCOs were cultured in 1 mL of media and treated with morphine daily. hCOs were treated with morphine on the final day of experiments and collected 3 h later for RNA extraction.

## HIV infection

hCOs were infected with NL4.3 HIV-1 EGFP BaL reporter virus using protocol previously established with minor modifications. First, hCOs were seeded in 96-well-plates and inoculated with 100 µl in OptiMEM containing diluted virus and Polybrene at a final concentration of 5 µg/ml. hCOs were subjected to spinoculation at 1200 g for 2 h at 32 °C, then placed in incubator at 37 °C for 6 h. Following incubation, inoculum was removed, hCOs were washed twice in PBS, plated in ultra-low attachment 24 well-plates, and incubated at 37 °C in 1mL complete hCO media on a continuous shaker.

## RT-qPCR

RNA was extracted using RNA extraction kit following manufacturer’s instructions (New England Biolabs). RT-qPCR for Pol and β-actin, as well as MOR-1 and β-actin, with custom HEX and FAM probes for each in the same reaction mixture using Luna^®^ Universal Probe One-Step RT-qPCR Kit (New England Biolabs) while RT-qPCR for HIV receptors CD4, CCR5, and CXCR4, with β-actin as a reference gene was performed using Luna^®^ Universal One-Step RT-qPCR Kit (New England Biolabs) as previously described (Donadoni et al. 2024). RT-qPCR reactions were performed in a LightCycler 96 instrument (Roche, Indianapolis, IN, USA) and the primers used for all qPCRs are listed in Table 1.

**Table 1:** List of primers and probes used

Gene	Primer name	Sequence (5’→3’)	Reference
CD4	Fw	TTCAGGACACAGGGAAATCAGGGTT	(Donadoni et al. 2024)
	Rv	GGAAGTGGTGAGGAAGGGTAGGAAG	
CCR5	Fw	TCTCTTCTGGGCTCCCTACAACATT	(Donadoni et al. 2024)
	Rv	TCTCTGTCACCTGCATAGCTTGGTC	
CXCR4	Fw	CTTTGTCATCACGCTTCCCTTCTGG	(Donadoni et al. 2024)
	Rv	AGGACACTGCTGTAGAGGTTGACTG	
β–Actin	Fw	CCTCGCCTTTGCCGATCC	N/A
	Rv	CGCGGCGATATCATCATCC	
Pol	Fw	ACAGACAATGGCAGCAATTTCACC	(Donadoni et al. 2024)
	Rv	TGCCAAATTCCTGCTTGATCCC	
	Probe-FAM	CGCCCACCAACAGGCGGCCTTAACTG	
MOR-1	Fw	CTCCACTCGAATTCGTCAGAAC	N/A
	Rv	GGCAACGGAGCAGTTTCT	
	Probe-HEX	CCTCCACGGCCAATACAGTGGAT	
β–Actin	Fw	GCATCCTCACCCTGAAGTA	(Donadoni et al. 2024)
	Rv	CACGCAGCTCATTGTAGAAG	
	Probe-HEX	ACCAACTGGGACGACATGGAGAAA	
	Probe-FAM	CACGCAGCTCATTGTAGAAG	N/A

### p24 Gag ELISA

NL4.3 HIV-1 EGFP BaL titer was measured by p24 Gag ELISA (Advanced BioScience Laboratories, Inc.), following instructions provided by the manufacturer. After HIV infection and morphine treatment of hCOs, 200uL supernatants were collected daily, and levels of HIV-1 viral load were also quantified by p24 Gag ELISA.

### RNAscope

hCOs were fixed in 4% PFA and embedded in OCT using methods previously established (Donadoni et al. 2024). The protocol of the RNAscope^®^ 2.5 HD Detection Reagents-RED assay (ACD™, 322360) was followed for the frozen sections of hCOs with modifications as previously described (Donadoni et al. 2024). Following RNAscope and cover slipping, slides were imaged with a Keyence microscope. Images were analyzed using ImageJ. HIV RNA dots and cells were identified, and data is graphed at dots/cell.

### Statistics

All the values on the graphs are presented as mean, and the standard error of the mean are reported as error bars on the histograms. Student’s *t* test and ANOVA were performed and p values < 0.05 were considered statistically significant. The raw data were analyzed by GraphPad Prism version 9.4.1.

## Data Availability

No datasets were generated or analysed during the current study.
